# Lithium chloride promotes proliferation of neural stem cells *in vitro*, possibly by triggering the Wnt signaling pathway

**DOI:** 10.1080/19768354.2018.1487334

**Published:** 2018-11-30

**Authors:** Jian Zhang, Lu He, Zhong Yang, Lihong Li, Wenqin Cai

**Affiliations:** a Department of Geriatrics, Chinese PLA 113rd Hospital, Ningbo, People’s Republic of China; b Department of Neurobiology, The Third Military Medical University, Chongqing, People’s Republic of China

**Keywords:** Lithium chloride, Wnt signaling pathway, β-catenin, GSK-3β, neural stem cells

## Abstract

The objective of this study was to clarify the relationship between the effect and associated mechanisms of lithium chloride on neural stem cells (NSCs) and the Wnt signaling pathway. The expression of key molecules proteins related to the Wnt signaling pathway in the proliferation and differentiation of control NSCs and lithium chloride-treated NSCs was detected by Western blot analysis. Flow cytometry analysis was applied to study the cell cycle dynamics of control NSCs and NSCs treated with lithium chloride. The therapeutic concentrations of lithium chloride stimulated NSC proliferation. β-catenin expression gradually decreased, while Gsk-3β expression gradually increased (*P *< 0.01). Furthermore, NSCs treated with lithium chloride showed significantly enhanced β-catenin expression and inhibited Gsk-3β expression in a dose-dependent manner. NSCs in the G0/G1-phases were activated with an increased therapeutic concentration of lithium chloride, while NSCs in the S-phase, as well as G2/M-phases, were arrested (*P *< 0.01). These data confirm that the proliferation of NSCs is remarkably promoted through changes of cell dynamics after treatment with lithium chloride. Our results provide insight into the effects of lithium chloride in promoting the proliferation abilities of NSCs *in vitro* and preventing the cells from differentiating, which is potentially mediated by activation of the Wnt signaling pathway.

## Introduction

The neural stem cells (NSCs) found in developing and adult brains are multipotent cells with the capacity for proliferation and differentiation into the primary cell types of the nervous system, such as neurons, astrocytes, and oligodendrocytes. These cells are essential for the repair of damaged brain tissues and the treatment of various neurodegenerative diseases (Cooper and Isacson 2004; Wang et al. 2015). Therefore, it is of great importance to discover critical pathways and targets that regulate the proliferation and differentiation of NSCs, as these are pressing concerns in the medical community (Blurton-Jones et al. 2009; Liu et al. 2014). NSC replacement therapy is a promising treatment for neurodegenerative disorders, including Parkinson’s disease (PD) and many others (Tincer et al. 2016; Liu et al. 2015). The ability to enhance the proliferation abilities and predetermine the fate of NSCs has the potential to increase the efficiency of cell-transplantation therapy for neurodegenerative diseases (Taupin 2006; Vazey and Connor 2010). NSCs provide a powerful and effective resource for cell-based transplantation therapies for the treatment of neurodegenerative diseases, yet the molecular mechanisms involved in the proliferation and differentiation of NSCs have not been elucidated (Somoza et al. 2010; Jang et al. 2015). We hypothesized that the Wnt signaling pathway may control the maintenance, proliferation, and neural differentiation of NSCs.

The canonical Wnt signaling pathway plays a vital role in controlling neurogenesis, NSC properties, and NSC differentiation (Hirabayashi et al. 2004; Wang et al. 2015). Wnt signaling activates this pathway, which leads to the accumulation of β-catenin in the cytoplasm. β-catenin is an essential effector molecule in the Wnt signaling pathway. GSK-3β antagonizes the Wnt signaling pathway, playing a central role in the development of diverse organisms including the central nervous system(CNS). Moreover, it is a downstream signaling component of the Wnt signaling pathway that regulates several developmental processes, including cell proliferation, apoptosis, migration, polarity, and fate decisions (Tiwari et al. 2015; Hari et al. 2002). Some evidence has suggested that the Wnt signaling pathway may also be involved in the neural differentiation of embryonic cells, neural progenitor cells, and neural stem cells (Otero et al. 2004; Prakash and Wurst 2007). However, several studies have shown that this pathway maintains NSCs in a proliferative state (Dastjerdi et al. 2012). During the mammalian embryonic development, the Wnt signaling pathway regulates the proliferation and cell fate of NSCs (Chenn and Walsh 2002; Zechner et al. 2003). The Wnt signaling pathway is implicated in the control of cell growth and differentiation during the development of CNS, but there is limited knowledge of its action at the cellular levels.

Lithium chloride, a mood-stabilizing drug that has been widely used in the treatment of bipolar affective disorder, also has dramatic effects on neurogenesis in the early development of the CNS (Korur et al. 2009). Lithium chloride acts through inhibition of glycogen synthase kinase-3β (GSK-3β), which regulates cell fate determination and neurogenesis in diverse organisms including CNS. Evidence has indicated that lithium chloride directly inhibits GSK-3β *in vitro* and it has been proposed that GSK-3β is an endogenous target of lithium chloride in these diverse systems (Klein and Melton 1996; Korur et al. 2009). Recent studies have demonstrated that lithium chloride has neuroprotective and neurotrophic properties, which may relate to its clinical effectiveness (Huo et al. 2012; Ishii et al. 2008). In addition, it was recently shown that therapeutic concentrations of lithium chloride significantly increases the proliferation and neuronal differentiation of neural progenitor cells *in vitro* (Su et al. 2009).

In this study, we aimed to investigate the effects of lithium chloride on cell viability, cycle dynamics, proliferation, and differentiation of NSCs derived from the cortex of rats *in vitro*, along with discovering its relationship with the Wnt signaling pathway. We hypothesized that the Wnt signaling pathway might be involved in the formation of neurospheres, stem cell maintenance, and proliferation of lithium chloride-induced NSCs. The results from this study may broaden our understanding of the neuroprotective effects of lithium chloride. In addition, this study may provide new insight into the mechanisms involved in the attenuation of neurodegeneration and the pathogenesis of lithium chloride in treating bipolar affective disorder in patients.

## Materials and methods

### Experimental animals

Healthy Sprague–Dawley (SD) rats that were pregnant for 14–16 days were provided by the Experimental Animal Center of the Third Military Medical University (Chongqing, China). The weights of the female SD rats ranged from 20 to 30 g. All procedures with animals were conducted in accordance with a protocol approved by the Experimental Animal Committee of the Third Military Medical University.

### Experimental drugs, reagents, and equipment

The following drugs and reagents were used: lithium chloride (Alfa Aesar B21573, Haverhill, MA, USA), β-catenin antibody (Santa Cruz C2206, Dallas, TX, USA), GSK-3β antibody (Santa Cruz AB15328), Nestin antibody (Abcam ab6142, Cambridgeshire, UK), MAP2 antibody (Merck Millipore AB5622, Burlington, MA, USA), GFAP antibody (Merck Millipore MAB3402C3), neurofilament antibody (Merck Millipore AB15328, MA), DMEM/F12 (Thermo Fisher, Waltham, MA, USA), basic fibroblast growth factor (bFGF, PeproTech, Rocky Hill, NJ, USA), epidermal growth factor (EGF, PeproTech), 0.25% trypsin (Sigma Aldrich, St. Louis, MO, USA), 0.01 mol/L RNase (Sigma Aldrich), 0.5 mg/L propidium iodide staining solution (Boster Biology, Wuhan, China), 5% Chloral hydrate (China), and 4% paraformaldehyde (Boster Biology). The equipment included a cold centrifuge (Sigma Aldrich, USA), an inverted light microscope (Olympus IX70, Tokyo, Japan), a microplate reader (Thermo Fisher), an incubator that was set at a temperature of 37°C and regulated with 5% CO_2_ (Thermo Fisher), and flow cytometer (FACSCalibur 2, Becton Dickinson, Franklin Lakes, NJ, USA).

### Culturing of NSCs from pregnant SD rats

Embryonic NSCs were collected from gestational 14–16 d old SD rats after an intra-abdominal injection of 30 mg/kg phenobarbital sodium. Cortices were harvested from two-thirds of the forebrain cortex of embryonic rat brains after removal of the meningeal tissue. Next, the fresh tissues were mechanically dissociated in cold D-Hank’s reagent, and the proliferation medium (DMEM/F12) was added. The cell suspension was gently mixed by pulling the solution up and down approximately 12–15 times using a pipette. After filtrating the cell suspension using filter mesh, the cell number of every culture bottle was adjusted to approximately 1×10^6^ cells/mL. A pipette was used to transfer the cells into every culture bottle, and the cells were maintained supplemented with DMEM/F12 + 20 uL/mL 50X B27 + 20 ng/ml bFGF prior to incubation. On the third day, half the volume of culture medium was transferred, and the cells were passaged between days six to nine with treatments beginning on day nine.

### Neurosphere assay and identification of NSCs

The neurosphere formation assay was performed to evaluate the long-term effects of lithium chloride on the maintenance and proliferation of NSCs. The neurospheres gradually formed and grew, with increasing cell numbers and sphere size after culturing for one week. After the lithium chloride treatment study, the neurospheres were imaged using an inverted light microscope. For identification of the NSCs, the cultured neurospheres were directly fixed with 4% paraformaldehyde in phosphate buffered saline (PBS) for 10 min and rinsed with PBS for immunocytochemistry. Next, the primary antibodies, including Nestin, MAP2, GFAP, and Neurofilament, were respectively added to the cells after blocking with 1% bovine serum albumin (BSA) for 1 h at 37°C. Fluorescein isothiocyanate (FITC)-anti-rabbit IgG was used to stain the nucleus. Fluorescence images of the cells were acquired using a confocal laser scanning microscope.

### Expression of Wnt signaling molecules during the differentiation of NSCs

To confirm a possible role of Wnt signaling molecules in the proliferation and differentiation of NSCs *in vitro*, we examined the expression of the Wnt signaling molecules. The NSCs were isolated and cultured from rat fetuses (age 14–16 days) according to previously reported methods. After extracting and culturing the NSCs for three passages, the cells were treated in media containing 10% fetal bovine serum (FBS) to promote differentiation. NSC cultures derived from the cortex of SD rats were carried out for the control, treated with FBS after 12, 24, 48, 72 h, the cells attached and grew processes gradually. Next, the cultured cells were freshly collected, and Western blotting was performed and quantified for the different groups.

### Effect of lithium chloride on the proliferation of NSCs

NSCs in the logarithmic growth phase were obtained and digested with trypsin for 5 min. The cell density was adjusted to 80 cells/µL. They were placed in the incubator with 5% CO_2_ at a constant temperature of 37°C. After 24 h, the supernatant was removed and the proliferation medium was added to the negative control group. Meanwhile, the four treatment groups and the control were established, which included lithium chloride treatments of 1, 5, 10, and 40 mmol/L. The treated cells were incubated for 3 d before being imaged using an inverted light microscope. The results were expressed as the degree of NSC proliferation. After the cultured cells were freshly collected, total protein was collected from each group and used for the Western blot.

### Western blotting

Western blotting was performed to quantify β-catenin and GSK-3β expression levels in the proliferation and differentiation process of normal NSCs and cultured NSCs treated with lithium chloride. Briefly, protein extracts were prepared from cell cultures of each group. The fresh samples were mechanically homogenized at 4°C in five volumes of extraction buffer and centrifuged for 10 min. After calculating the total protein, the supernatant was mixed with 4X protein loading buffer, boiled for 5 min at 99°C, and stored at 4°C. Total protein was loaded for electrophoresis on 10% denaturing PAGE gels and transferred to a nitrocellulose membrane. The membranes were blocked with 5% skim milk in Tris-buffered saline containing 0.05% Tween 20, followed by incubation with the β-catenin or GSK-3β primary antibodies and corresponding secondary antibodies. The bands were visualized and quantified using the Quantity One 4.6.2 program.

### Detection of cell cycle dynamics

Flow cytometry analysis was used to measure the cell cycle dynamics in different cell phases of the cell cycle. After the application of lithium chloride for 72 h, the cultured NSCs were collected and digested with 0.25% trypsin for 15 min at 37°C. Next, a 100 µm mesh sieve screening was performed before the cells were fixed with 75% ethanol at 4°C. The cell suspension was treated with 100 uL RNase at 37°C for 30 min, washed with PBS, and stained with 0.5 mg/L propidium iodide staining solution for 30 min at 4°C in the dark. The stained cells were analyzed using a FACSCalibur cell analyzer and software. Each experiment was performed five-times or more.

### Statistical analysis

SPSS 20.0 software (IBM, Chicago, IL, USA) was used for statistical analysis and experimental data were expressed as the mean ± SD. The statistical significance of the differences was analyzed using the Student’s *t*-test, and *p*-values <0.05 were considered statistically significant.

## Results

### Culture and immunofluorescence identification of NSCs

**Figure 1.**
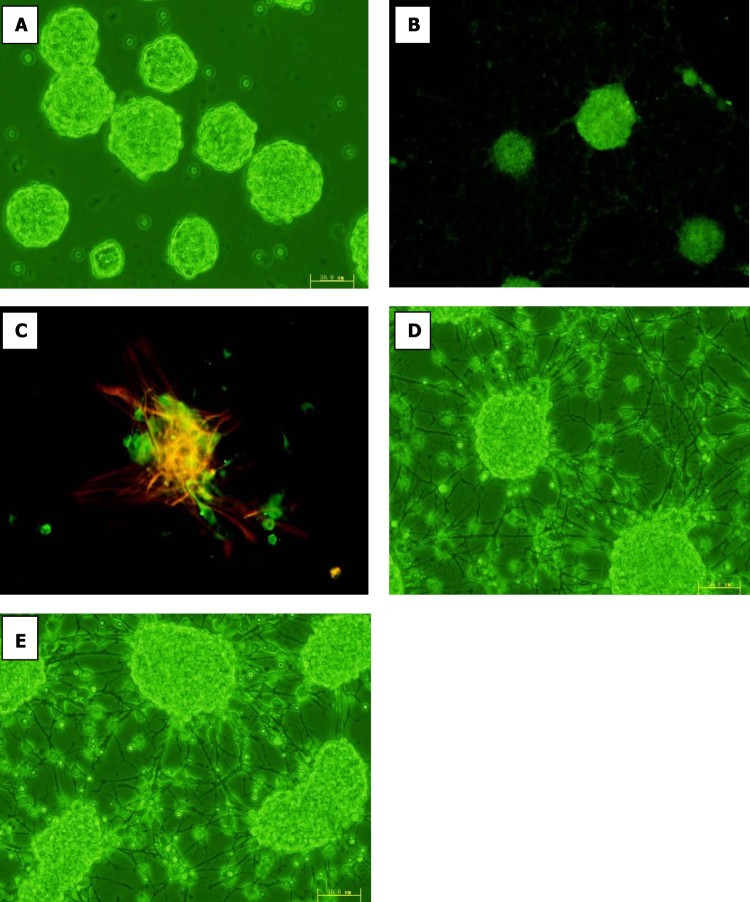
Immunofluorescence identification of NSCs. (A) The culturing NSCs after 3 days were observed floating, NSCs(neurosphere) culture derived from rat cortex. (×200) (B) Immunofluorescence technique was applied to detect the signal of Nestin antigens of the culturing NSCs. The Nestin^+^ (neural stem cell marker) cells are labeled in green. (Anti-Nestin FITC ×200) (C) Immunofluorescence technique was applied to detect the signal of Map2 antigens and GFAP antigens of the culturing NSCs after 7 days treated in media containing 10% fetal bovine serum (FBS).The differentiation marker Map2^+^ (neuron)cells are labeled in green, whereas the GFAP^+^ (astrocyte) cells are shown in red, and Hoechst counterstaining is shown in yellow. (Anti-MAP2 FITC/ Anti-GFAP Cy3 ×200) (D,E) The culturing NSCs were observed after 48 h treated in media containing 10% fetal bovine serum (FBS). (×200).

For the preparation of NSCs, fresh tissues were dissected from the forebrain cortex of pregnant rat embryos. On the third day of subculturing the NSCs, the neurospheres had grown to suitable sizes after three passages and suspended growth of neurospheres was notably observed (Figure 1). In the confocal dish coated with collagen, better adherent growth was observed for the NSCs. The neurosphere formation assay was performed because neurosphere formation is directly correlated with the self-renewal of NSCs. Furthermore, characterization of the cell culture was performed by immunocytochemistry with various neural antibodies. Immunostaining revealed that neurospheres stained positive for Nestin. In addition, the NSC populations were characterized using Nestin staining (Figure 1). The cell proliferation and differentiation abilities of NSCs were confirmed by MAP2 and GFAP incorporation and Nestin labeling experiments (Figure 1). These data indicate that cultured NSCs have proliferative and differentiative abilities. Our results provide a valuable resource for studying the self-renewal, proliferation, and differentiation of NSCs.

### Role of Wnt signaling molecules during the proliferation and differentiation of cultured NSCs

**Figure 2.**
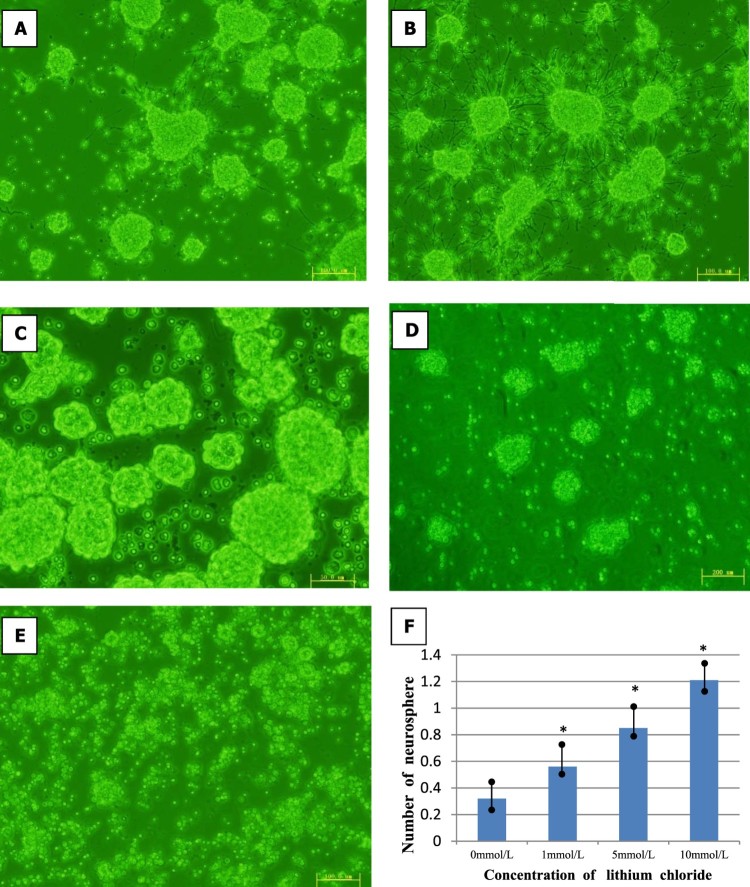
Western blot showing dynamic changes of GSK-3ß and ß-catenin levels in the differentiation of the culturing NSCs treated in media containing 10% fetal bovine serum (FBS). (A) Immunoblots of GSK-3ß(94KD) and ß-catenin(94KD) in control and FBS group at 12 h, 24 h, 48 h and 72 h. (B) Comparison of GSK-3ß(94KD) and ß-catenin(94KD) levels in these groups. ANOVA shows significance: ^※^*P *< 0.05, ^※※^*P *< 0.01 vs. control(mean ± SD, *n* = 4).

Differentiation of the neurospheres was investigated in the presence of 10% FBS, under which conditions the NSCs attached and gradually grew. The NSCs, derived from the SD rat cortexes were treated with 10% FBS after 12 h, 24 h, 48 h, or 72 h, were shown to differentiate into neurons, oligodendrocytes, and astrocytes (Figure 1). The expression of the primary components involved in the Wnt signaling pathway, including β-catenin and GSK-3β, was detected in NSCs *in vitro* via Western blot analysis, This indicates that *in vitro* NSCs can respond to Wnt signaling pathway during cell differentiation processes. It was found that after being treated with 10% FBS for 12 h to 72 h, β-catenin and GSK-3β were found to be persistently expressed during differentiation. Interestingly, the signal density of the β-catenin protein in the NSCs gradually decreased, while that of GSK-3β gradually increased, when compared with the control (Figure 2). These data indicate that the expression of Wnt signaling molecules is an essential modulator during neural proliferation and differentiation of NSCs.

### Involvement of the Wnt signaling pathway in the lithium chloride-induced survival and proliferation of NSCs

**Figure 3.**
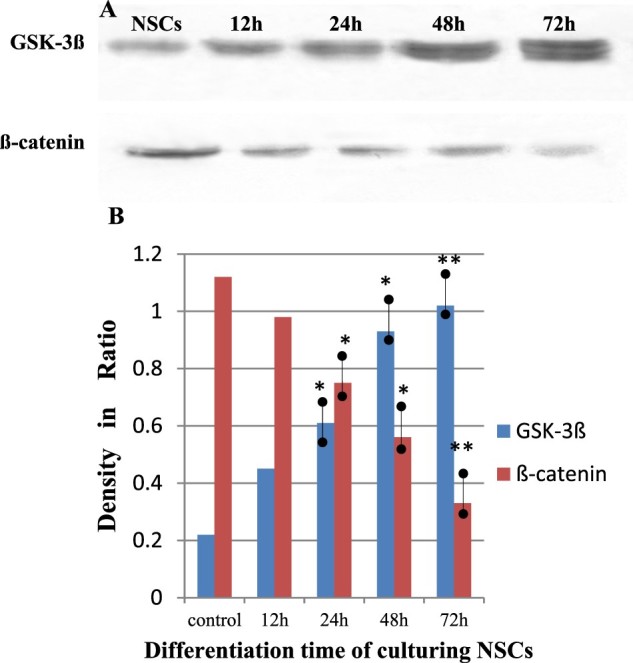
The expression of GSK-3ß and ß-catenin protein levels after cultured NSCs treated with Lithium chloride. Cells were cultured in vitro for 2 days, then exposed to different concentrations of Lithium chloride (1 mmol/L, 5 mmol/L, 10 mmol/L) for another 3 days. (A) Immunoblot analysis of GSK-3ß and ß-catenin in four groups of the culturing NSCs treated with different concentration lithium chloride. Western blot results showed lithium chloride –triggered dynamic changes of GSK-3ß and ß-catenin level in the culturing NSCs. (B) Comparison of GSK-3ß and ß-catenin protein levels in these groups of normal NSCs and the lithium chloride treated group. Relative quantification of Western blot analysis is depicted in the bar graphs. Data were shown as mean ± SD. ^※^*P *< 0.05, ^※※^*P *< 0.01 versus control(NSCs).

Considering that Wnt signaling molecules play essential roles in the neural proliferation and differentiation of NSCs *in vitro*, we further explored whether the stimulating effect of lithium chloride was attributable to its regulation of the Wnt signaling pathway. In this study, lithium chloride was used to inhibit GSK-3β and to activate β-catenin for the treatment of NSCs. Given that lithium chloride may exert cytotoxic or protective effects on cells, which depends on the concentration used for treatment, it is imperative to ascertain a safe concentration range of lithium chloride. To this end, the NSCs were exposed to lithium chloride at the concentrations ranging from 1 to 10 mmol/L. Western blot analysis further confirmed that lithium chloride increased β-catenin protein expression, while higher concentrations of lithium chloride gradually suppressed GSK-3β levels in the treatment groups (1 to 10 mmol/L lithium chloride treatment), as shown in (Figure 3). Lithium chloride activated the Wnt signaling pathway, as revealed by the up-regulation of β-catenin expression and substantially decreased expression of GSK-3β in a dose-dependent manner.

**Figure 4.**
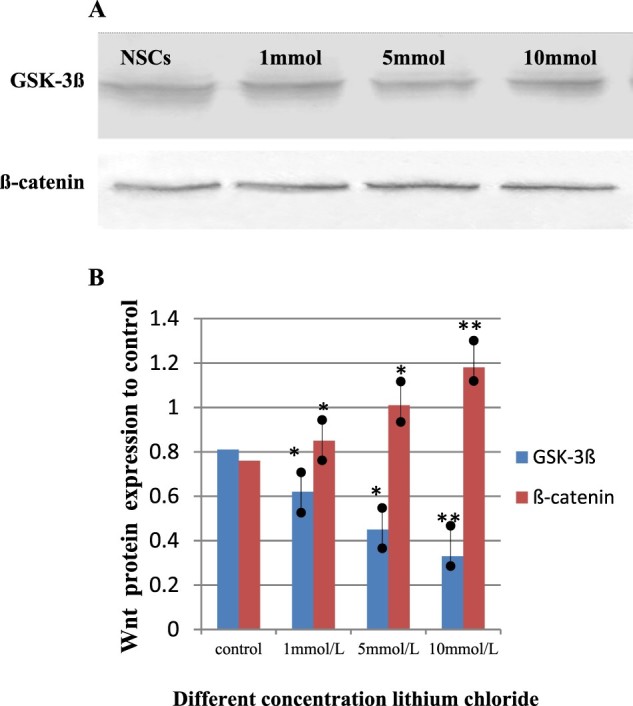
Effects of lithium chloride on NSCs proliferation and neurosphere formation. (A,B) The culturing NSCs were observed after 48 h treated in media containing 10% fetal bovine serum (FBS) and treated with lithium chloride. (×200) (C) The culturing NSCs were observed after 3days treated with 1 mmol lithium chloride. (×200) (D) The culturing NSCs were observed after 3days treated with 5 mmol lithium chloride. (×200). (E) The culturing NSCs were observed after 3days treated with 10 mmol lithium chloride. (×200) (F) Cells were exposed to different concentrations of lithium chloride(1,5,10,40 mmol) for 3 days. Neurosphere formation was checked under a microscope, then the numbers of neurosphere in different groups were analyzed. Data were shown as mean ± SD. *P *< 0.05 versus control.

**Figure 5.**
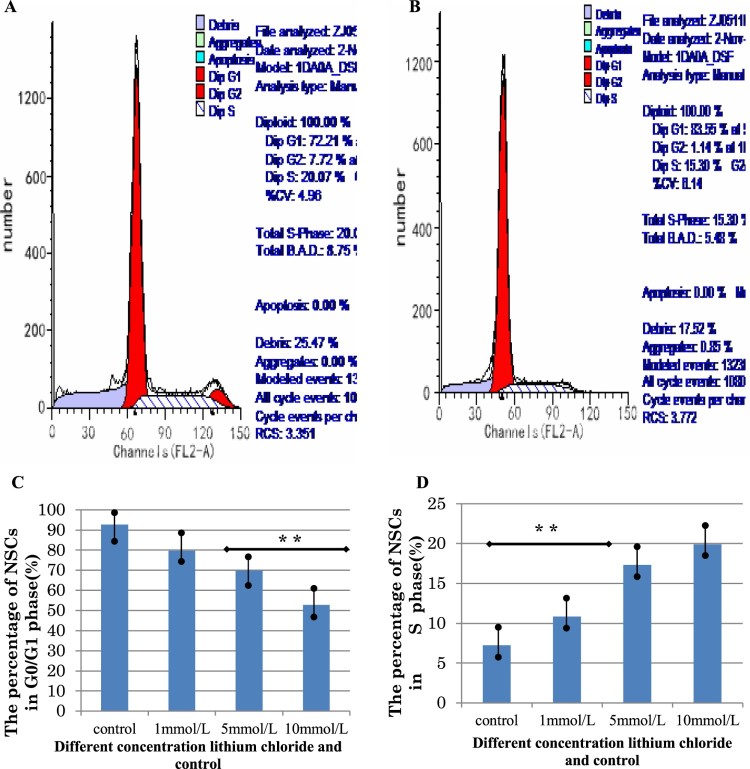
The influence of Lithium chloride on the cell cycle dynamics and proliferation of NSCs. (A,B) Flow cytometric cell cycle profiles of NSCs suspension in the normal control group and the 10 mmol/L lithium chloride treated group. (C) The percentage of NSCs in G0/G1 phase is depicted in the bar graphs, respectively. The data express means ± SD. ^※^*P *< 0.05, ^※※^*P *< 0.01 versus control. (D) The percentage of NSCs in S phase is depicted in the bar graphs, respectively. The data express means ± SD. ^※^*P *< 0.05, ^※※^*P *< 0.01 versus control.

In the cultured NSCs neurospheres, administration of lithium chloride stimulated the proliferation of NSCs in a dose-dependent manner. At the concentrations of 1, 5, 10, and 40 mmol/L, lithium chloride significantly increased the size and number of neurosphere. Compared with the control group, NSCs proliferation significantly increased in the lithium chloride groups after the Wnt signaling pathway was activated (Figure 4). Next, flow cytometry analysis was used to study the influence of lithium chloride on cell cycle dynamics and proliferation of NSCs *in vitro*. The results showed that treatment for 3 d with lithium chloride accompanied an increase in NSC arrest (G0/G1-phase) when compared with the controls (*p *< 0.01, Figure 5). However, the percentage of NSCs in the S-phase and G2/M-phase was higher than that of the controls (*p *< 0.01, Figure 5). We confirmed that the proliferation of NSCs was remarkably increased by changing the cell cycle dynamics with lithium chloride treatment in a dose-dependent manner. These results suggest that lithium chloride promotes the proliferation, survival, and stem cell maintenance of NSCs *in vitro* by triggering the Wnt signaling pathway.

## Discussion

NSCs are a powerful resource for cell-based transplantation therapies. However, it is essential to understand the molecular mechanisms by which NSC proliferation and differentiation occurs in order to identify new therapeutic approaches for treating brain damage and neurodegenerative diseases. β-catenin and GSK-3β, as major effector molecules in the canonical Wnt signaling pathway, can regulate the proliferation, expansion, and differentiation of NSCs (Cui et al. 2011; Ahn et al. 2008). Lithium chloride, a psychiatric drug widely used to treat bipolar affective disorder, remarkably inhibits GSK-3β. A potential link between lithium chloride and the Wnt signaling pathway was recently discovered, yet the differential involvement of this signaling remains undiscovered (Kim et al. 2004; de Melker et al. 2004). In this study, we discovered a new potential Wnt signaling pathway mechanism in mediating the stimulating effects of lithium chloride on NSCs. Our results demonstrate that lithium chloride can effectively promote NSC proliferation, survival, and stem cell maintenance by regulating pivotal molecules involved in the Wnt signaling pathway.

Our data provide a valuable resource for studying the survival, proliferation, and self-renewal properties of NSCs. After isolating, culturing, and identifying the NSCs, we observed that the functioning of Wnt signaling molecules during the proliferation and differentiation of NSCs *in vitro*. These results suggest that the expression of Wnt signaling molecules is an important modulator in the neural proliferation and differentiation of NSCs. Compared with the control group, lithium chloride significantly increased β-catenin levels and effectively activated the Wnt signaling pathway, while the expression of GSK-3β was reduced in a dose-dependent manner. Meanwhile, flow cytometry analysis showed that lithium chloride remarkably promoted NSC proliferation through cell cycle alterations (dose-dependent). These results suggest that NSCs can not only trigger the Wnt signaling pathway, but also promote the proliferation, expansion, and stem cell maintenance of NSCs, after treatment with lithium chloride. In addition, lithium chloride may also play a neuroprotective role for NSCs through this pathway.

In summary, lithium chloride conferred impressive and long-term neuroprotective effects by triggering the Wnt signaling pathway and promoting NSC proliferation and survival (Li et al. 2011). From the *in vitro* cell culture models, which can recapitulate many of the primary cellular processes found during nervous system development, lithium chloride evaluated for the treatment of CNS disorders and neurodegeneration diseases, such as Huntington disease, Alzheimer disease, dementia, severe depression, and bipolar affective disorder (Vazey and Connor 2010; Kulkami and Dhir 2010; Aubert et al. 2002). Combined with the current experimental results, we hypothesize that lithium chloride may exert a neuroprotective effect through the Wnt signaling pathway.

Furthermore, the Wnt signaling pathway plays an important role in controlling the proliferation and differentiation of NSCs (Prakash and Wurst 2007; Bengoa-Vergniory et al. 2014). Activation of the Wnt signaling pathway effectively reversed the diminished proliferative and neuronal or dopaminergic differentiation potential of NSCs (L’Episcopo et al. 2014; Lie et al. 2005). Reduced oxygen levels are beneficial for NSC cultures, as it leads to enhanced proliferation, survival, and differentiation potential, which is correlated with the activation of the Wnt signaling pathway (Braunschweig et al. 2015). It has also been confirmed that brain-derived neurotrophic factors may contribute to proliferation and neuronal differentiation of NSCs *in vitro* possibly by triggering the Wnt/β-catenin signaling pathway (Chen et al. 2013). In the last few years, there have been strong interests in the role that Wnt signaling pathway plays in controlling NSC proliferation and stem cell fate (Kriska et al. 2016; Jang et al. 2015). For this reason, understanding the mechanisms involved in the regulation of the Wnt signaling pathway is of critical importance, especially in the survival, maintenance, differentiation of stem cells. In our experiment, Western blot and flow cytometry analyses showed that Wnt signaling plays critical roles in the proliferation and differentiation of NSCs. When combined, these data demonstrate that the Wnt signaling pathway can promote the proliferation, survival, and stem cell maintenance of NSCs through the regulation of β-catenin protein level. Consequently, our findings suggest that the Wnt signaling pathway possibly supports the role of lithium chloride in activating NSC proliferation. Furthermore, triggering of the Wnt signaling pathway is a feasible approach for the treatment of neurodegenerative diseases.

NSC replacement therapy is a promising treatment for neurodegenerative disorders, including PD. For this reason, a variety of strategies have been proposed in the treatment of neurodegenerative diseases by triggering the Wnt signaling pathway (Moon et al. 2004; Chen et al. 2005; Han et al. 2017). It has been confirmed that *in vitro* priming of adult NSCs with lithium chloride may augment cell transplant efficiency and confer impressive long-term protection against neonatal hypoxia-ischemia (Vazey and Connor 2010; Li et al. 2011). As a Wnt signaling molecule agonist, lithium chloride has a therapeutic role in the treatment of neurodegenerative diseases and acute brain injuries (Wade et al. 2005). Using NSC culture systems *in vitro*, we demonstrate that lithium chloride is an essential factor regulating the proliferation and stem cell maintenance of NSCs via activation of the Wnt signaling pathway. These data provide insight into the mechanisms by which lithium chloride provides neurorestorative effects and suggests a potential new strategy for treatment of neurodegenerative diseases. Collectively, our findings potentiate lithium chloride as a promising activator of the Wnt signaling pathway to promote the proliferation, survival, and stem cell maintenance of NSCs. Studies have indicated that transient exposure to lithium chloride, during *in vitro* proliferation of neural progenitor cells, can alter the differential fate and increase the proportion of neuron cells (Qi et al. 2017; Vazey and Connor 2009). In future experiments, we will study the neuronal differentiation effect of NSCs treated with lithium chloride. In addition, we will continue to study other pathways in order to better understand the mechanisms of action for lithium chloride on NSCs. This study provides experimental evidence for the rational and efficient use of lithium chloride as a satisfactory treatment strategy with NSC replacement therapy for treating neurodegenerative diseases. We hope that novel clinical manipulation strategies targeting the Wnt signaling pathway in cell replacement therapies will be developed for the treatment of neurodegenerative diseases in the future.

## Conclusion

In summary, we demonstrated that lithium chloride could effectively promote the proliferation of NSCs by triggering the Wnt signaling pathway. Lithium chloride functions by up-regulating the Wnt signaling pathway, which provides an excellent target for the development of new treatments for those neurodegenerative diseases that may be characterized by NSC proliferation. It provides exciting prospects that lithium chloride might be a new novel therapeutic targeting NSCs for the treatment of neurodegenerative diseases and bipolar affective disorder. Further clinical studies are warranted to confirm the neuroprotective effects of lithium chloride in patients.
